# Male–female interactions drive the (un)repeatability of copula duration in an insect

**DOI:** 10.1098/rsos.160962

**Published:** 2017-02-22

**Authors:** Paul E. Eady, Denise V. Brown

**Affiliations:** 1 School of Life Sciences, University of Lincoln, Lincoln LN6 7TS, UK; 2 Faculty of Applied Sciences, University of Sunderland, Sunderland SR1 3SD, UK

**Keywords:** sexual selection, sperm competition, cryptic female choice, callosobruchus

## Abstract

Across the animal kingdom the duration of copulation varies enormously from a few seconds to several days. Functional explanations for this variation are largely embedded within sperm competition theory in which males modulate the duration of copula in order to optimize their fitness. However, copulation is the union of two protagonists which are likely to have separate and often conflicting reproductive interests, yet few experimental designs specifically assess the effect of male–female interactions on the duration of copulation. This can result in inexact assertions over which sex controls copulatory behaviour. Here we analyse the repeatability of copulatory behaviour in the seed beetle *Callosobruchus maculatus* to determine which sex exerts primary influence over copulation duration. In *C. maculatus*, copulation follows two distinct phases: an initial quiescent phase followed by a period of vigorous female kicking behaviour that culminates in the termination of copulation. When males or females copulated with several novel mates, copulatory behaviour was not significantly repeatable. By contrast, when males or females mated repeatedly with the same mate, copula duration was repeatable. These data suggest copulatory behaviour in *C. maculatus* to be largely the product of male–female interactions rather than the consistent, sex-specific modulation of copula duration of one protagonist in response to the phenotypic variation presented by the other protagonist.

## 1. Introduction

The duration of copulation varies enormously from a few seconds in the yellow fever mosquito (*Aedes aegypti*) [1] to over 10 days in the southern green stink bug (*Nezava viridula*) [2]. Functional explanations for this variation are largely embedded within sperm competition theory [3], such that males modulate the duration of copula in order to optimize fitness via sperm pre-emption and anti-sperm pre-emption mechanisms [4]. However, females also have a vested interest in the duration of copulation. For example, they may bias paternity in favour of particular males by varying the duration of copula in response to cues delivered by males during copulation [5,6], or they might vary the period of time spent in copula in order to optimize time allocated to other activities such as foraging, egg laying, avoiding predation and/or avoiding injury [7–9]. Thus, there are good reasons to expect females to exert some control over the duration of copulation, which may well be in conflict with the fitness optima of the male protagonist [10].

Sexual conflict over mating and copulation is well documented in the bruchid beetle *Callosobruchus maculatus*. Males have spines on their genitalia that puncture the female reproductive tract during copulation. The level of damage inflicted on females is positively related to the duration of copulation [9,11] and negatively related to female fitness ([9,11–13], although see [14]). Copulation in *C. maculatus* is characterized by two phases. In phase I, the male and female remain relatively sedentary following genital coupling, while in phase II the female vigorously kicks at her mate with her hind legs [15]. Ablation of the female's hind legs prevents females from directly kicking their mates and this results in an extended copulation duration and subsequently greater levels of genital damage [16]. This had led to the suggestion that females kick at their mates in order to reduce the duration of copulation and thus the level of harm sustained [16].

Why males prolong copula and by extension inflict greater genital damage on their mates' remains unclear. Rönn & Hotzy [13] found no evidence that the spines function as an anchor to prevent the dislodgement of copulating males by rivals, while Edvardsson & Canal [16] found no evidence that extended copulation benefited males via its effects on female re-mating propensity or male success in sperm competition (see also [11]). Hotzy *et al*. [17] reported that males with longer spines had enhanced success in sperm competition. However, they also found no evidence that spine length correlated with copulation duration, thus the relationship between spine length, copulation duration and extent of genital damage remains to be fully resolved.

That there are reproductive conflicts between the sexes in this and other species [10,18] has led a number of authors to seek which sex controls copulation duration [19–25]. However, teasing apart male and female influences over the duration of copulation is difficult. For example, pairings with large females often elicit longer copulations [26,27] which is usually interpreted as males strategically investing more ejaculate to large, fecund females (i.e. cryptic male choice) [27]. However, in comparison to small females, larger females tend to live longer [14], lay more eggs [24] and have larger sperm storage organs [28] thus, it may well be in their best interests to receive more sperm via an extended duration of copulation. One approach to circumvent these problems of interpretation is to partition variation in copulation duration between males and females that are derived from different strains (or geographical locations). In a series of intra- and inter-strain mating's involving *Drosophila mojavensis*, Krebs [26] was able to show that both male and female population origin affected the duration of copulation, with male population origin explaining five times more of the variation in copulation duration than female population origin. However, while this approach clearly partitions variation in copulation duration to male and female population origin, it does not tell us which sex exerts primary influence over copula duration within a population.

Here we examine the repeatability of copulation duration in order to identify which, if any, sex has a predominant role in determining the length of copulation in the bruchid beetle *C. maculatus*. The logic follows that if males primarily determine the duration of copulation they will modulate their behaviour according to the phenotype of the female. Thus, when the same female phenotype is presented to different males, copulation duration of the female should be repeatable (have a high intra-class correlation coefficient [29]). Should females exert primary control over copulation then the opposite holds; different females presented with the same male phenotype should have similar (repeatable) copulatory characteristics. By contrast, should copulation duration be driven by a male–female interaction, the repeatability of copulation duration when males and females are presented with different mates will be low.

## 2. Material and methods

The *C. maculatus* used here were originally from Niamey, Niger and at the time of the experiments had been in laboratory culture for approximately 50 generations. Stock cultures were maintained on black-eyed beans (*Vigna unguiculata*) at 27°C, approximately 35% relative humidity and a 16 L : 8 D photoperiod. All beetles used in the experiments described below were initially virgin and between 24 and 48 h from eclosion at their first copulation. All matings and behavioural observations took place at 27°C. Under laboratory conditions about 80% of females remate within 48 h of an initial copulation so long as an adequate oviposition resource is available [30]. Female remating is also influenced by male mating status: virgin males induce longer inter-mating refractory periods in females than do non-virgin males [31].

To determine the effect of the male, the female and male–female interactions on the duration of copulation, three treatments were established: females mated repeatedly to different males, males mated repeatedly to different females, and males/females mated repeatedly to the same partner. In all treatments, focal individuals were mated four times with copulations separated by a 48 h interval, during which females laid eggs on 25 black-eyed beans. In all treatments, the mating history of the focal individual's mate was controlled such that the focal individual's 2nd, 3rd and 4th mates had previously mated once, twice and thrice, respectively. Copulation was observed under individual 50 ml Perspex pots and recorded as: (i) time from genital coupling until the female kicks at the male, and (ii) time from the onset of female kicking to genital disengagement [32]. To assess the effect of male and female size on copulation duration, male and female elytra lengths were measured under an Olympus SZH stereomicroscope linked to an image analysis workstation.

The effects of male and female identity on copula duration were assessed in a single classification ANOVA in which male (or female) identity were entered as a random independent variable and the duration of copula (and its phases) entered as the dependent variable. Intra-class correlations coefficients and their 95% confidence intervals (CIs) were calculated using the ICC package in R [33]. The duration of two of the measures of copulatory behaviour (time from genital coupling to the onset of female kicking and total duration of copula) increased with mating order (see Results and [34]). Thus, in these cases, repeatability was calculated from values normalized around the mean for each mating order, within each treatment (treatment being either copulating with the same mate or copulating with different mates).

## 3. Results

**Figure 1.**
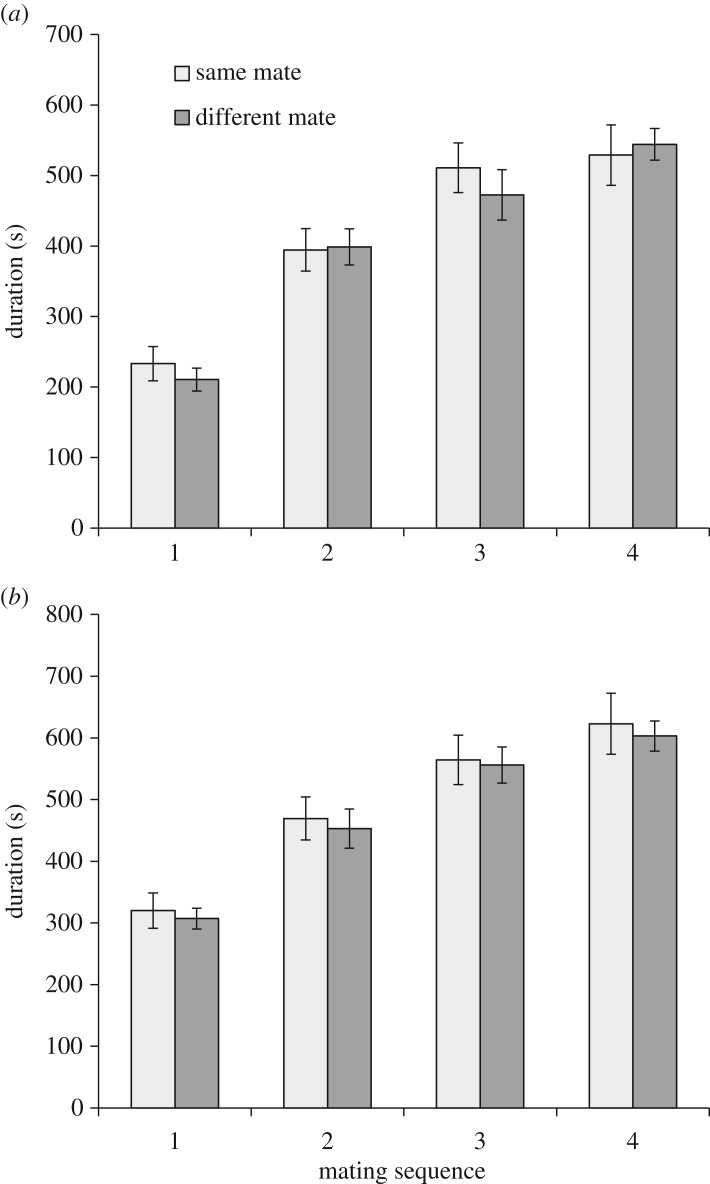
Mean (±s.e.) duration (seconds) of (*a*) genital coupling to the onset of female kicking and (*b*) total copulation, in relation to both mating sequence and treatment (i.e. whether copulation was with the same or different mates).

The duration of the start-to-kick phase of copulation increased with mating order irrespective of whether females were mated repeatedly to the same male or different males (two-way ANOVA: treatment (same or different male) *F*_1,141_ = 0.24, *p* = 0.62; mating order *F*_3,141_ = 44.9, *p* < 0.0001; figure 1*a*). Thus, subsequent analyses of repeatability were carried out on normalized values. When females mated to the same male on four separate occasions the duration of the start-to-kick phase was repeatable (*F*_19,55_ = 2.72, *p* = 0.002, intra-class correlation coefficient = 0.31, 95% CI 0.58–0.09). However, when females were mated to different males or males mated to different females, the duration of the start-to-kick phase was not significantly repeatable (*F*_19,54_ = 1.53, *p* = 0.11, intra-class correlation coefficient = 0.13, 95% CI 0.40−0.03 and *F*_19,54_ = 0.81, *p* = 0.69, intra-class correlation coefficient = 0.06, 95% CI 0.19−0.19, respectively). The duration of the start-to-kick phase was not related to male size, female size or the interaction between male and female size in both the same-mate and different-mate treatments (*p* > 0.13 in all cases).

Mate sequence had no effect on the duration of the kick-to-end phase of copulation (two-way ANOVA: treatment (same or different male) *F*_1,141_ = 0.08, *p* = 0.77; mating order *F*_3,141_ = 0.8, *p* = 0.49), thus intra-class correlation coefficients were calculated on the actual (i.e. non-normalized) data. When females mated to the same male on four separate occasions the duration of the kick-to-end phase of copulation was repeatable (*F*_19,55_ = 2.97, *p* = 0.001, intra-class correlation coefficient = 0.34, 95% CI 0.61–0.12). However, when females were mated to different males or males mated to different females, the duration of the kick-to-end phase was not significantly repeatable (*F*_19,54_ = 0.89, *p* = 0.60, intra-class correlation coefficient = 0.03, 95% CI 0.22−0.18 and *F*_19,54_ = 1.61, *p* = 0.09, intra-class correlation coefficient = 0.14, 95% CI 0.41−0.06, respectively). The duration of the kick-to-end phase was not related to male size, female size or their interaction in both the same-mate and different-mate treatments (*p* > 0.34 in all cases).

A similar pattern emerges from the analysis of total copulation duration. As with the duration of the first phase of copulation, total copulation duration increased with successive matings (two-way ANOVA: treatment (same or different male) *F*_1,141_ = 0.39, *p* = 0.53; mating order *F*_3,141_ = 32.8, *p* < 0.0001; figure 1*b*). Analyses of the normalized copulation duration values revealed the total duration of copulation to be repeatable when females mated to the same male on four separate occasions (*F*_19,55_ = 4.32, *p* < 0.0001, intra-class correlation coefficient = 0.47, 95% CI 0.70–0.24). However, when females were mated to different males the intra-class correlation coefficient was marginally non-significant (*F*_19,54_ = 1.77, *p* = 0.052, intra-class correlation coefficient = 0.17, 95% CI 0.45−0.03) and when males mated to different females the intra-class correlation was non-significant (*F*_19,54_ = 0.52, *p* = 0.94, intra-class correlation coefficient = −0.15, 95% CI 0.05−0.25). Total copulation duration was not related to male size, female size or their interaction in both the same-mate and different-mate treatments (*p* > 0.14 in all cases).

## 4. Discussion

Here we have shown copulation duration in the seed beetle *C. maculatus* to be largely the product of a male–female interaction. Neither males nor females were consistent in their copulatory behaviour when paired with different mates at each copulation. This lack of repeatability could arise from males and females adjusting their behaviours in response to cues derived from dynamic internal states and external stimuli such that each copulatory event represents a unique set of circumstances to both interacting parties. For a male, salient external stimuli might include female status (virgin or not), female fecundity (size), the likelihood of finding another mate or the likelihood of facing sperm competition [4,27], while females might vary the duration of copulation in response to male quality [5,6] and/or time constraints associated with other activities, such as finding suitable oviposition sites. Some of these external stimuli could be conveyed via the hydrocarbon profiles of both males and females as these are known to vary ontogenetically and in response to social cues [35]. They have also been shown to affect the duration of copula in *Drosophila melanogaster* [36]. However, apart from male and female quality, external factors were held constant across the same-mate/different-mate treatments, plus there was no evidence that absolute or relative mate size affected the duration of copula. Thus, had males strategically invested more ejaculate in larger females, we would have expected to observe consistently longer copulations when males were paired with large females. Low levels of copulation duration repeatability have been previously reported for the Queensland fruit fly (*Bactrocera tryoni*) when males copulated with different mates (*r* < 0.1; [23,37]).

That copulation duration is a product of a male–female interaction also makes sense from a functional perspective as under a polyandrous mating strategy the two sexes are likely to have different fitness optima associated with time spent in copula [6,10] and are thus likely to evolve traits that enable them to shape the duration of copula towards their own sex-specific optima. Which sex wins this conflict appears to depend on the value of winning and power (the relative contest costs of the two sexes [38]). Males typically have higher values of winning [39] but power may be loaded in favour of the female (especially in invertebrates where females are typically larger than males). Thus, we expect copulation duration to be influenced by male and female traits. Despite this, a number of studies point to male or female control over the duration of copulation. For example, Crickmore & Vosshall [40] show that copulation duration in *D. melanogaster* is under the control of the male (see also [41]), which is primarily driven by the interplay between GABAergic interneurons of the male abdominal ganglion and dopaminergic neurons of the ventral nerve cord. By contrast, females appear to exert control over copulation duration in *Drosophila montana*. In this species copulation appears to bear many similarities to that observed in *C. maculatus* in that the initial stages of copulation appear harmonious while the latter stages are characterized by vigorous kicking at the mounted male by the female. Males copulating with dead females (i.e. no female kicking) engaged in copula that was substantially longer than those copulating with normal females [42]. Similar experiments in the Queensland fruit fly (*B. tryoni*) also point to female control (or at least influence) over copulation duration [23] as do the results of leg ablation experiments in female *C. maculatus* [16].

The low repeatability of copulation duration would indicate low heritability [43]. Despite this, several studies report heritable variation in the duration of copulation in insects [44–46] including *C. maculatus* [12,20,47]. Low repeatability and significant heritable variation could result from different studies using different populations (heritability is a population specific measure) or different experimental approaches. For example, Savalli & Fox [20] and Brown *et al*. [47] estimated the heritability of copulation duration based on matings between virgin adults, whereas here we estimated repeatability when individuals were either virgin (1st mating) or non-virgin (subsequent matings). Similar inconsistencies exist in the *Drosophila* literature: Gromko [44] found copulation duration to be heritable in *D. melanogaster* while Taylor *et al.* [48] found no evidence for heritable variation in this behaviour and suggested this was owing to the inherent phenotypic and genetic variability of the females used to assay the copulation duration of focal males.

That copulation duration increases with copulatory sequence (see also [34]) suggests copulation duration is negatively related to the size of the ejaculate transferred in this species. This is based on the well-established observation that *C. maculatus* males transfer smaller ejaculates [49] and fewer sperm [31] at each successive mating. Similar results can be inferred from the studies of Ofuya [50] and Fox *et al*. [49] which show older males to both spend longer in copula and transfer smaller ejaculates than younger males, and Vasudeva *et al*. [51] who found males exposed to thermal stress during development transferred fewer sperm but spent longer in copula than males reared under optimal conditions. Taken together, these studies indicate copulation duration to be negatively related to male quality in *C. maculatus* and could account for the negative phenotypic and genotypic association between copulation duration and longevity found in this species [47]. However, the relationship between copulation duration and ejaculate size is clearly a complex one as Edvardson & Canal [16] and van Leishout *et al*. [52] report ejaculate weight to increase with increasing copulation duration, while Savalli & Fox [20] and Brown *et al*. [47] found no relationship. The upshot of these conflicting results is that caution should be exercised when interpreting the adaptive value of extended copulation without detailed knowledge of its relationship with sperm and ejaculate transfer.

## Data accessibility

Datasets associated with this study are available at http://dx.doi.org/10.5061/dryad.b0h4h [53].
